# Association Analysis of *GABRA5*, *SOX13*, and *AGL* Gene Polymorphisms with Growth Traits in Dongfeng Sika Deer

**DOI:** 10.3390/biology15110881

**Published:** 2026-06-03

**Authors:** Yan Zhang, Xinyuan Zhang, Huansheng Han, Xue Wang

**Affiliations:** College of Animal Science and Veterinary Medicine, Heilongjiang Bayi Agricultural University, Daqing 163319, China; zhangyan990625@163.com (Y.Z.); zhangxinyuan011015@163.com (X.Z.); wangxuedonglin@126.com (X.W.)

**Keywords:** Dongfeng sika deer, growth traits, haplotype, molecular marker, correlation analysis

## Abstract

To investigate the association between polymorphisms in the *GABRA5*, *SOX13*, and *AGL* genes and growth traits in Dongfeng sika deer and to identify potential molecular markers for breeding, this study was conducted based on prior genome-wide association analysis. Based on the previous GWAS analysis of 266 Dongfeng sika deer, the SNP loci of *GABRA5*, *SOX13*, and *AGL* genes were detected in 36 male deer samples. The genetic parameters were calculated, and an association analysis with growth traits was carried out. Phenotypic analysis indicated that body weight and chest circumference had higher coefficients of variation than other growth traits, and body weight showed a strong positive correlation with body-slant length (r = 0.743, *p* < 0.01) and a moderate correlation with chest circumference (r = 0.709). A total of six SNP loci were identified, including three within *GABRA5* (Chr13-8442730, Chr13-9033380, and Chr13-9045819), one within *SOX13* (Chr14-5681678), and two within *AGL* (Chr20-66603370 and Chr20-66618510). The dominant genotypes at these loci include CG (CC), AA, CG, CC (CG), AA, and GG (GC). Linkage disequilibrium analysis revealed a relatively strong association between Chr13-8442730 and Chr13-903380 on chromosome 13. Combined genotype analysis showed that diplotype CCCGGC was associated with higher body weight and larger chest circumference than other genotype combinations. Gene expression analysis showed that the relative expression levels of *GABRA5*, *SOX13*, and *AGL* were lower in the low-growth group than in the high-growth group, and expression variation was also observed within groups. Overall, gene expression levels appeared to be positively associated with growth traits, with higher expression associated with improved growth performance. These findings suggest that *GABRA5* and *AGL* may serve as candidate genes for further investigation and that the identified SNP loci may contribute to the development of molecular markers for the selection of growth traits in Dongfeng sika deer. The results provide a preliminary basis for molecular breeding and genetic improvement strategies in Dongfeng sika deer bucks and serve as an important reference for genetic improvement of growth traits in Cervidae.

## Simple Summary

This study aimed to identify molecular markers associated with growth traits in male Dongfeng sika deer by analyzing the polymorphisms and expression patterns of *GABRA5*, *SOX13*, and *AGL* genes. A total of six SNPs were detected in the three genes, all showing moderate polymorphism and conforming to the Hardy–Weinberg equilibrium. Significant associations were found between these SNPs and body weight and chest circumference, with the diplotype CCCGGC identified as the superior genotype combination for enhanced growth performance. Quantitative real-time PCR revealed that the mRNA expression levels of all three genes were significantly higher in the high-growth group than in the low-growth group, indicating a positive correlation with growth traits. These findings suggest that *GABRA5* and *AGL* are promising candidate genes, and the identified SNPs provide effective molecular markers for molecular breeding to improve growth traits in Dongfeng sika deer.

## 1. Introduction

Gamma-aminobutyric acid type A receptor alpha 5 subunit (*GABRA5*) is a key component of inhibitory neurotransmission within the central nervous system, where it contributes to the fine regulation of neuronal excitability and synaptic inhibition. The *GABRA5* gene [1] has been reported to be involved in the inhibitory modulation of the hypothalamic appetite center and the regulation of energy expenditure. The *GABRA5* gene may act in concert with *ERC2*, *FHIT*, and other genes, providing a potential clue to the genetic basis of growth traits in sika deer, potentially through a coordinated “neural–metabolic–cellular” regulatory network. Butler et al. [2] have demonstrated that *GABRA5* and *GABRA2* play key roles in nervous system development and the regulation of neuronal excitability, providing direct evidence for the mechanistic study of development-related diseases. The SRY-related HMG-box (SOX) gene family is a class of transcription factors containing highly conserved HMG domains, which widely regulate biological processes such as embryonic development, organogenesis, and cell differentiation [3,4,5]. Among them, SOX13, as an important member of the family, participates in the processes of liver metabolic maturation and cartilage and development and plays a key role in body growth and metabolic regulation [6,7]. Amylo-alpha-1, 6-glucosidase, 4-alpha-glucanotransferase (*AGL*) has both starch 1, 6-glucosidase and 4α-glucotransferase activities; maintains glucose homeostasis by regulating glycogen metabolism; and plays an important role in energy metabolism and growth and development [8,9,10,11,12]. Studies have confirmed that *AGL* gene polymorphism is significantly associated with pig growth and carcass traits [13] and can be used as a potential molecular marker for livestock and poultry growth selection, suggesting that *AGL* gene polymorphism also has important research value in the regulation of growth traits in deer animals.

After years of breeding and directional cultivation, Dongfeng sika deer have formed a genetically stable population. This deer caste is homozygous, hereditary performance is stable, disease resistance is good, tolerance is strong, and production performance is excellent. In 2004, it passed the new breed certificate of the Ministry of Agriculture of China [14]. Building on prior genome-wide association analyses conducted in our laboratory, three candidate genes—namely, *GABRA5*, *SOX13*, and *AGL*—were identified among six SNP loci associated with bodyweight and chest circumference traits [15]. Despite these preliminary findings, there are no reports on the polymorphism of *GABRA5*, *SOX13*, and *AGL* genes in sika deer. Given their documented or inferred roles in neurodevelopmental regulation, metabolic processes, and growth-related phenotypes, it is plausible that genetic variation within *GABRA5*, *SOX13*, and *AGL* may contribute to phenotypic variability in growth traits in Dongfeng sika deer, although this relationship requires empirical validation. Accordingly, the present study utilized whole-genome resequencing data to identify six SNP loci within the *GABRA5*, *SOX13*, and *AGL* genes in Dongfeng sika deer bucks, followed by an assessment of the genetic characteristics of the population and association with key growth traits. Blood, as a relatively easy and low-damage experimental sample to animals, can reflect the metabolism of animals, while blood transcriptome can further reflect the physiological metabolic state and molecular mechanism of animals and is widely used to understand the expression of host genes, which is helpful in understanding the function and structure of genes at the overall level and in revealing specific biological functions [16]. This study aimed to provide a theoretical basis for further verification of the functions of *GABRA5*, *SOX13*, and *AGL* genes and to identify effective molecular markers for molecular breeding of sika deer.

## 2. Materials and Methods

### 2.1. SNP and Growth-Trait Data Acquisition

The whole-genome resequencing data and phenotypic records analyzed in this study were derived from previously published work by our group [15], with the cohort extended to include additional individuals generated under comparable experimental conditions. In total, 302 Dongfeng sika deer bucks in clinically healthy condition were included, comprising 266 previously reported individuals and 36 newly included animals. All animals were managed under the same breeding and management conditions. Phenotypic measurements were obtained in accordance with the Technical Specifications for Determination of Antler Deer Production Performance (NY/T1179-2006 [17]), with all measurements conducted by the same trained surveyor to reduce inter-observer variability. Using a measuring rod and tape, ten quantitative traits were recorded, including body weight (WT), body-slant length (BL), body height (HT), chest circumference (WS), chest depth (CC), head length (HL), frontal width (FW), horn-stalk distance (JB), tube circumference (PC), and tail length (WC). Following quality control and filtering, clean reads were aligned to the red deer reference genome (Cervus elaphus, assembly mCerEla 1.1, annotated version Release 100), after which SNPs were identified and subjected to genotype imputation and filtering and high-confidence SNPs were retained for downstream analyses.

### 2.2. PCR Amplification and Sequencing

The primer design for the six SNP loci within the *GABRA5*, *SOX13*, and *AGL* genes was based on reference sequences obtained from the NCBI database, and primers were generated using the Primer 3 Plus (https://www.primer3plus.com/) online platform. The resulting primer sequences (Table 1) were synthesized by GenScript Biotechnology Co., Ltd. (Nanjing, China) and stored at 4 °C.

Genomic DNA samples extracted from Dongfeng sika deer were pooled in equimolar concentrations to generate mixed DNA templates, which were subsequently used for PCR amplification of target gene fragments. PCR amplification was carried out in a total reaction volume of 25 μL, comprising 1 μL each of forward and reverse primers (10 μmol/L), 3 μL of DNA template, 12.5 μL of 2× SanTaq PCR Mix (Model: B532061; Sangon Biotech Co., Ltd., Shanghai, China, containing MgCl_2_, dNTPs, Taq DNA Polymerase, PCR buffer, and PCR enhancers), and 7.5 μL of deionized water. The PCR amplification procedure included an initial denaturation step at 94 °C for 5 min, followed by 30 cycles of denaturation at 94 °C for 30 s, annealing at 55–65 °C for 30 s, and extension at 72 °C for 45 s, with a final extension at 72 °C for 10 min and subsequent holding at 4 °C. Amplified products were verified using 1% agarose gel electrophoresis, and PCR products meeting quality criteria were submitted to Heilongjiang Jiansu Gene Technology Co., Ltd.(Harbin, Heilongjiang, China) for sequencing.

### 2.3. Preparation and Amplification of the qPCR Reaction System

Total RNA from venous blood of 6 sika deer was extracted by a Sangon Biological RNA Extraction Kit (Model B518653; Sangon Biotech Co., Ltd., Shanghai, China). Reverse transcription was subsequently performed using a HyperMB Rapid Reverse Transcription Kit (Model B690033; Vazyme Biotech Co., Ltd., Nanjing, China). The reaction procedure consisted of the following: reaction at 37 °C for 15 min followed by incubation at 85 °C for 15 s. The obtained cDNA was stored at −20 °C for reserve. Quantitative PCR was conducted using ChamQ Universal SYBR qPCR Master Mix (Model Q711; Vazyme Biotech Co., Ltd., Nanjing, China) in a 20 μL reaction system containing 2 μL of cDNA template, 10 μL of 2× master mix, 1 μL each of forward and reverse primer, and 6 μL of DEPC-treated water (Table 2). Amplification was performed on an Archimed X4 real-time PCR system (Kunpeng Gene Scientific Instrument Co., Ltd., Beijing, China) under the following cycling conditions: initial denaturation at 95 °C for 3 s, followed by 40 cycles of 95 °C for 10 s and 60 °C for 30 s.

### 2.4. Genotype and Allele Calculation

The calculation formula is genotype = the number of individuals of the genotype/the total number of samples of the determined population; allele = allele homozygous genotype + this gene heterozygous genotype/2.

### 2.5. Hardy–Weinberg Equilibrium Test

The Hardy–Weinberg equilibrium is a population in which genes and genotypes remain constant and in a stable equilibrium state across multiple generations (independent of specific interference factors, such as non-random mating, selection, migration, mutation, or limited population size). The chi-square statistic for HWE testing is calculated as follows:

(1) $$\chi^{2}=\sum \frac{(O_{i}-E_{i}{)}^{2}}{E_{i}}$$

where *O_i_* represents the observed genotype counts and *E_i_* represents the expected genotype counts. The calculated χ^2^ value was compared with the critical value from the chi-square distribution table. A locus was considered to conform to the Hardy–Weinberg equilibrium when *p* > 0.05 and considered to deviate from the Hardy–Weinberg equilibrium when *p* < 0.05.

### 2.6. Data Statistics

Genotype data for the six SNP loci were organized using Microsoft Excel 2019, and the distribution of dominant genotypes across the population was assessed to identify loci with low representation, which were subsequently excluded from combined analyses. The remaining SNP loci were integrated to construct genotype combinations, and individuals were grouped accordingly. The population was classified into different genotype combinations, and the significance of growth traits across these combinations was tested using one-way analysis of variance (ANOVA) and multiple comparisons (LSD). Six SNP loci of *GABRA5*, *SOX13*, and *AGL* genes were analyzed by Linkage Disequilibrium (LD) and Haplotype (Haplotype) analysis with the help of Haploview 4.2 software. The Pearson correlation method was used to analyze the correlation between different genotype combinations and chest circumference; qRT-PCR results were expressed as mean ± standard error (mean ± SEM), while the coefficient of variation (CV) was calculated as CV = (Standard deviation/Mean) × 100%, analyzed using the 2^−ΔΔCt^ method and plotted in GraphPad Prism 10.8.

## 3. Results

### 3.1. Determination and Correlation Analysis of Growth Traits of Dongfeng Sika Deer Bucks

Descriptive statistics for growth-related traits in 266 Dongfeng sika deer bucks are presented in Table 3. The standard deviations of growth size traits were small, with the exceptions of body weight and chest circumference, indicating that the degree of variation of growth size traits of Dongfeng sika deer bucks was relatively stable. The coefficients of variation ranged from 4.71% to 22.26%, with body weight (22.26%) exhibiting the highest variability. This pattern may suggest that this trait retains greater scope for genetic improvement under selective breeding.

Correlation analysis among growth traits of Dongfeng sika deer bucks (Table 4) revealed multiple statistically significant positive relationships. Body weight showed strong positive correlations with body length, body height, chest circumference, frontal width, and pipe circumference (*p* < 0.01) and more moderate associations with chest depth (*p* < 0.05). Body length was positively correlated with body height, frontal width, pipe circumference, and antler shank distance (*p* < 0.01). Body height demonstrated significant positive associations with chest circumference, head length, frontal width, and pipe circumference (*p* < 0.01) and a weaker but still significant relationship with antler shank distance (*p* < 0.05). Additional correlations were observed between head length and both frontal width and pipe circumference (*p* < 0.05), as well as between frontal width and pipe circumference (*p* < 0.01). Among them, the strongest association was observed between body weight and chest circumference (r = 0.855), followed by that between body weight and body length (r = 0.744).

### 3.2. Principal Component Analysis Results

**Figure 1:**
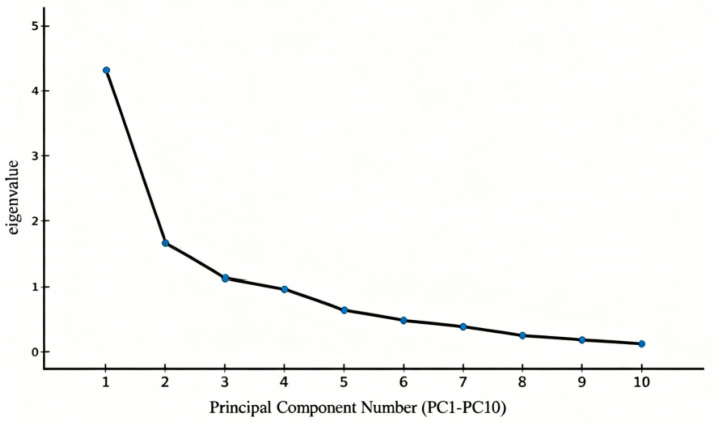
Scree plot of principal component analysis. Note: Scree plot showing the eigenvalues (Y-axis) of the first 10 principal components (X-axis) derived from 10 growth-related traits (body weight, body length, body height, chest circumference, chest depth, head length, frontal width, antler shank distance, tail length, and pipe circumference). Each principal component represents a linear combination of these growth traits rather than a single individual trait.

Principal component analysis results are shown in Figure 1. The scree plot exhibits a characteristic declining trend, and the first three principal components yielded eigenvalues of 4.296, 1.649, and 1.121, respectively, each exceeding the threshold value of 1.0 defined by Kaiser’s criterion. Beyond the third component, the eigenvalue curve approaches a plateau, indicating a diminishing explanatory contribution and a clear inflection point. According to the total-variance explanation table, the first three principal components accounted for 70.657% of the total variation, fully representing the main information structure of the original data. Therefore, this study retains the first three principal components for subsequent population structure analysis and GWAS covariate correction to eliminate the impact of population stratification on association results and improve their accuracy.

### 3.3. Genome-Wide Association Analysis Results

Genome-wide association analysis of 10 growth traits in Dongfeng sika deer bucks was performed using the GEMMA model, and the SNP loci associated with some growth traits are shown in Table 5. Following genotype quality control using PLINK (v1.90), a total of 27,127,458 SNPs were retained for analysis. Across all traits, 774 SNP loci reached the predefined significance threshold (−log_10_*P* = 7.5). Notably, candidate genes including *GABRA5* (chromosome 13), *SOX13* (chromosome 14), and *AGL* (chromosome 20) were identified among loci associated with both body weight and chest circumference, supporting their potential involvement in growth-related biological processes in this population.

### 3.4. Screening of Genotype Combinations at SNP Loci

Genomic DNA extracted from 36 individuals was assessed by 1% agarose gel electrophoresis. The observed bands were distinct, with no evidence of smearing or degradation, indicating that the DNA samples were of sufficient quality and integrity for downstream molecular analyses.

**Figure 2:**
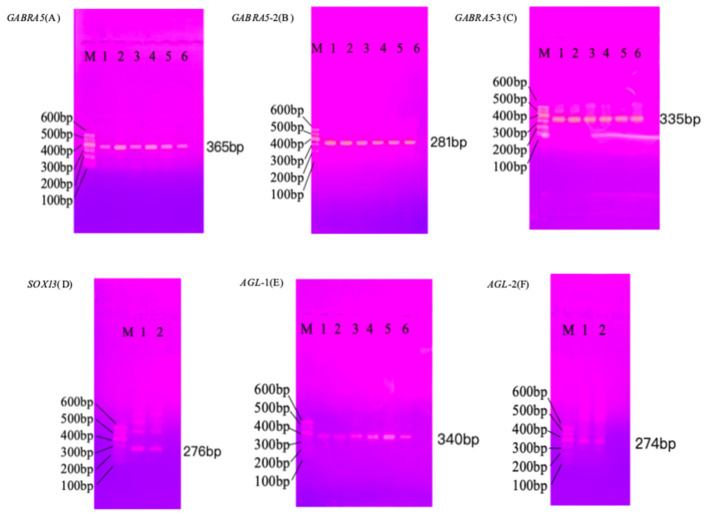
Agarose gel electrophoresis diagram. Note: *GABRA5*, *GABRA5-2*, *GABRA5-3*, *SOX13*, *AGL-1*, and *AGL-2* amplified products.

**Figure 3:**
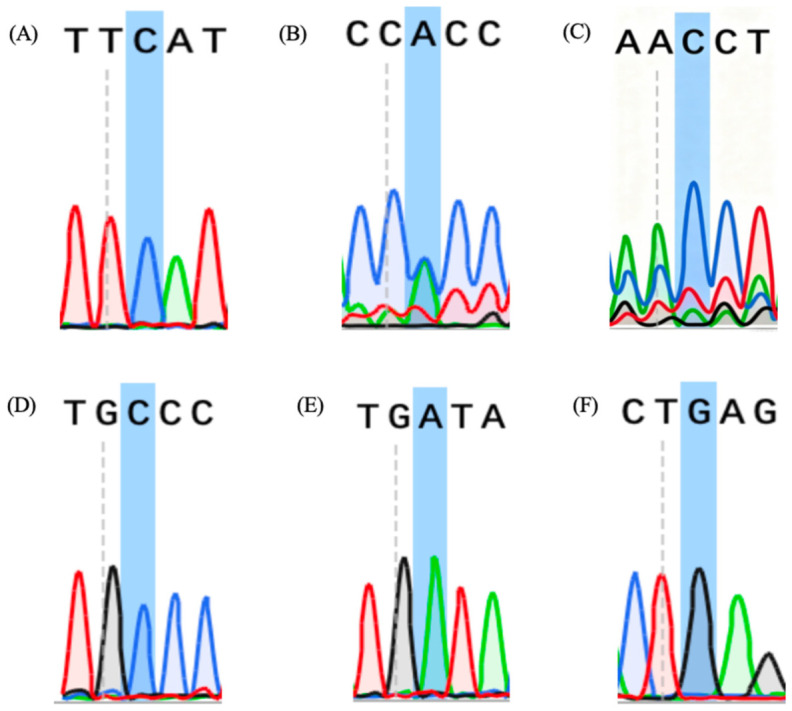
PCR amplification product sequencing. Note: (**A**) *GABRA5*, (**B**) *GABRA5-2*, (**C**) *GABRA5-3*, (**D**) *SOX13*, (**E**) *AGL-1*, and (**F**) *AGL-2* amplification product sequencing.

In this study, the PCR amplification and sequencing of six SNP loci of three candidate genes were successfully completed as shown in Figure 2 and Figure 3 and Figures S1–S6. The genetic diversity of the selected sika deer populations was analyzed, and the results are presented in Table 6 and Table 7. Observed heterozygosity (Ho) was consistently lower than expected heterozygosity (He), with mean values of 0.259 and 0.349, respectively, suggesting a modest deficit of heterozygotes in the sampled population. Polymorphic information content (PIC) values ranged from 0.142 to 0.375, with an average of 0.2795, consistent with moderate polymorphism. The highest PIC value was observed at SNP 14-5681678 (0.375), whereas the lowest was recorded at SNP 13-9045819 (0.142). The minimum allele frequency ranged from 0.083 to 0.500, with an average of 0.259. The effective number of alleles (Ne) ranged from 1.087 to 1.670, with an average of 1.308. Expected heterozygosity ranged from 0.153 to 0.500, while observed heterozygosity ranged from 0.083 to 0.500. Hardy–Weinberg equilibrium analysis indicated that all six SNP loci conformed to equilibrium expectations (*p* > 0.05).

### 3.5. Relationship Between SNP Loci and Growth Traits

Association analysis between SNP genotypes and growth traits was conducted in the subset of 36 genotyped individuals, with results summarized in Table 8. Significant differences (*p* < 0.05) were observed among genotypes at each locus for both body weight and chest circumference. For body weight, the dominant genotypes were CC, CG, and GC. Individuals with the CC genotype at SNP 13-8442730 had higher values than those with the CG genotype, whereas at SNP 14-5681678, the CG genotype was associated with greater body weight than the CC genotype. Similarly, at SNP 20-66618510, individuals with the GC genotype showed higher body weight than those with the GG genotype. For chest circumference, the dominant genotypes were CG, AA, CG, CC, AA, and GG. Genotype-specific differences were also evident: at SNP 13-8442730, CG individuals exceeded CC; at SNP 13-9033380, AA exceeded AT; at SNP 13-9045819, CG exceeded CC; at SNP 14-5681678, CC exceeded CG; at SNP 20-66603370, AA exceeded AT; and at SNP 20-66618510, GG exceeded GC. Collectively, these patterns suggest that heterozygous genotypes may be advantageous at certain loci associated with body weight, whereas homozygous genotypes are more frequently associated with increased chest circumference, though these trends are not uniform across all loci.

### 3.6. Haplotype Analysis

**Figure 4:**
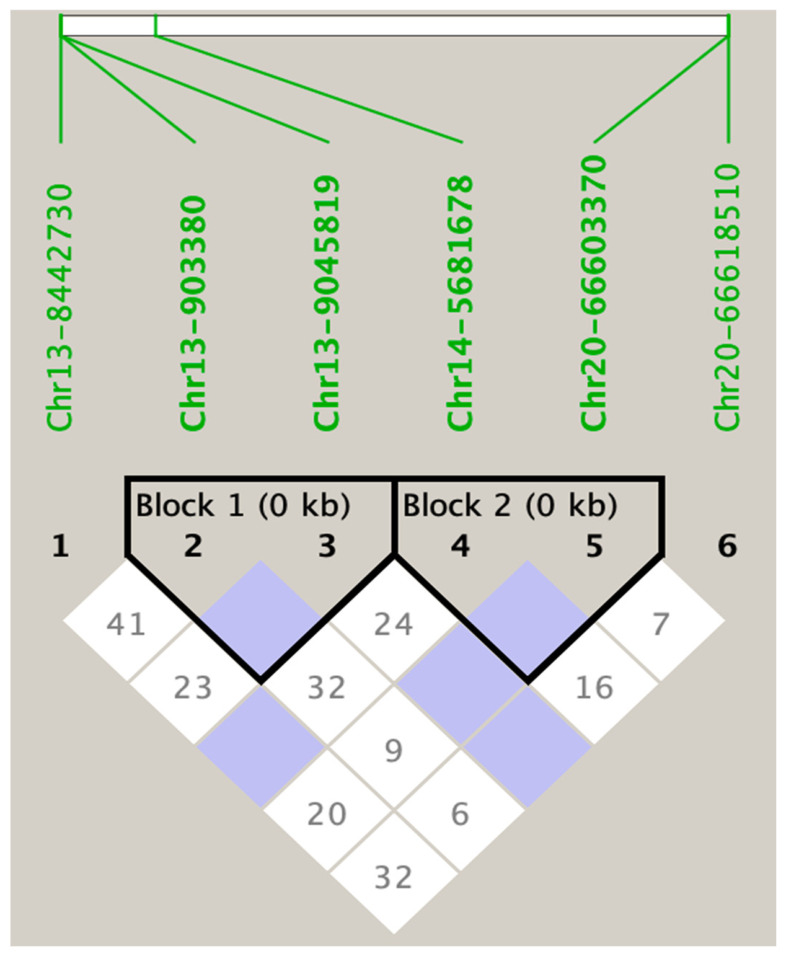
Analysis of linkage disequilibrium of *GABRA5*, *SOX13*, and *AGL* gene SNPs. Note: The colors of the linkage disequilibrium heatmap squares show linkage disequilibrium between SNP sites, with light colors representing complete linkage. Note: The color gradient of LD heatmap squares indicates the degree of linkage disequilibrium between SNP loci. The upper triangle represents D’ values, and the lower triangle represents r^2^ values.

Linkage disequilibrium (LD) analysis of the six SNP loci was performed using Haploview 4.2, as shown in Figure 4, and the haplotype frequencies of the six SNPs of *GABRA5*, *SOX13*, and *AGL* genes are shown in Table 9. Among loci located on chromosome 13 within the *GABRA5* gene, SNP pairs Chr13-8442730 (SNP1) and Chr13-903380 (SNP2) exhibited moderate LD (r^2^ = 0.41), while Chr13-903380 (SNP2) and Chr13-9045819 (SNP3) showed slightly lower LD (r^2^ = 0.32). Other intra-chromosomal associations were comparatively weaker. Inter-chromosomal LD values (across chromosomes 13, 14, and 20) were consistently below 0.33, indicating limited linkage and supporting the assumption of independent assortment, consistent with Mendelian expectations. In summary, the overall linkage disequilibrium among the six loci is low, and marker independence is good, making them suitable independent genetic markers for the association analysis of subsequent growth traits.

### 3.7. Effects of Different Genotype Combinations on Growth Traits

Combined genotype analysis was conducted using three SNP loci (SNP 13-8442730, SNP 14-5681678, and SNP 20-66618510), with genotype groupings presented in Table 10. Individuals were divided into three categories based on the number of dominant genotypes: Type I (0 dominant genotypes; *n* = 4; 11.11%), Type II (1–2 dominant genotypes; *n* = 27; 75.20%), and Type III (3 dominant genotypes; *n* = 5; 13.8%). The corresponding diplotypes for each category are specified in the tables. Significant differences in both body weight and chest circumference were observed among these groups, following the trend of Type III > Type II > Type I, indicating that the accumulation of favorable genotypes may be associated with enhanced growth performance.

### 3.8. Quantitative Fluorescence Analysis (qRT-PCR)

**Figure 5:**
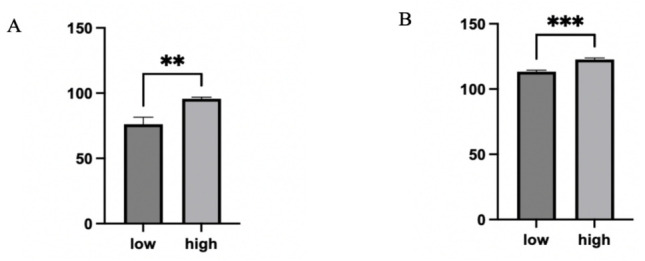
Body weight (**A**) and chest circumference (**B**) of Dongfeng sika deer in different groups. Note: ** represents a very significant difference (*p* < 0.01); *** Represents a very significant difference (*p* < 0.001).

**Figure 6:**
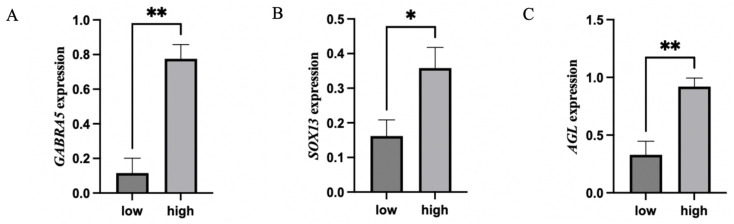
Expression of genes of *GABRA5* (**A**), *SOX13* (**B**), and *AGL* (**C**) in different groups. Note: * represents a significant difference (*p* < 0.05); ** represents a very significant difference (*p* < 0.01).

**Figure 7:**
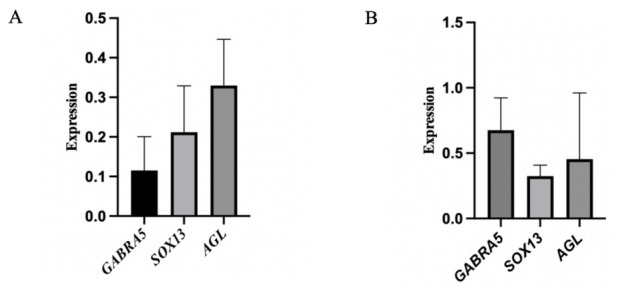
Expression of different genes in the same groups. (**A**) Expression of *GABRA5*, *SOX13*, and *AGL* in bodyweight group; (**B**) expression of *GABRA5*, *SOX13*, and *AGL* in bust circumference group.

In this study, six Dongfeng sika deer bucks were divided into high- and low-weight groups based on body weight and chest circumference; descriptive statistics are shown in Table 11. The candidate genes (*GABRA5*, *SOX13*, and *AGL*) selected in this study were analyzed by real-time fluorescent quantitative PCR, with the results shown in Figure 5, Figure 6 and Figure 7. Relative gene expression levels were derived from Ct values using the comparative quantification approach. The qRT-PCR validation showed that the relative expression levels of *GABRA5*, *SOX13*, and *AGL* were consistently lower in the low-phenotype group than in the high-phenotype group. This pattern suggests a positive association between the expression of these genes and growth-related traits, although the limited sample size necessitates cautious interpretation. Within each phenotypic group, differential expression among the three genes was also observed, with *AGL* exhibiting lower expression levels than *GABRA5* and *SOX13*. Notably, *GABRA5* expression was more pronounced in the high group, suggesting a stronger association with the phenotypic traits under investigation.

## 4. Discussion

In animal husbandry, selecting individuals with faster growth rates, improved body composition, and superior meat quality remains central to producing high-quality meat. Body weight and body size, as important phenotypic characteristics of livestock and poultry, play a key role in animal breeding and serve as indicators of animal production performance. These traits are shaped by the combined influence of genetic and environmental factors and therefore represent complex quantitative characteristics of substantial economic importance in livestock production systems [18]. In addition to providing a direct measure of body size and structural development, bodyweight and body size traits may also indirectly reflect the growth status of internal organs and physiological systems [19,20,21]. However, a considerable number of studies have examined these traits in conventional livestock species, including cattle [22], sheep [23], pigs [24], and chickens [25]; relatively few studies have focused on deer. Existing work in cervids has largely concentrated on the relationship between velvet antler traits and body size or weight, with limited emphasis on the direct association between body size and weight in deer species [26,27,28].

The polymorphic information content (PIC) is a key index to evaluate genetic variation in a population. PIC > 0.5 is highly polymorphic, 0.25 < PIC < 0.5 is moderately polymorphic, and PIC < 0.25 is low polymorphism. The PIC of the six SNP loci in this study ranged from 0.142 to 0.345, and the overall polymorphism was moderate. Expected heterozygosity (He) and observed heterozygosity (Ho) can reflect the level of genetic diversity in a population. In this study, the average He was 0.349, and the average effective allele number (Ne) was 1.308. There is a big difference between the estimated allele number and the actual allele number. The balance of allele distribution is general, and the overall genetic diversity is at a medium to low level. According to the Hardy–Weinberg equilibrium test, all loci conform to genetic equilibrium, and the genetic structure of the population is stable. This study further compared genetic diversity parameters such as the Ho, He, and PIC of Dongfeng sika deer with the genome-wide results of Ba et al. [29] based on simplified genome sequencing of sika deer in Northeast China. On the whole, the genetic diversity trends of the two are basically the same, and both show moderate genetic diversity, indicating that the overall genetic variation of sika deer farmed in Northeast China is relatively rich, with potential for molecular marker-assisted breeding. Compared with the whole-genome background, some functional candidate gene loci in this study have a lower He, presuming that growth-related functional genes are more affected by artificial targeted breeding and purification selection. Both studies confirmed that there was no significant inbreeding decline in the sika deer population and that the genetic structure was stable. The genetic diversity of the population in this study is slightly low, which may also be related to the single genetic background of the breeding population, the fixed breeding direction, and the minimal introduction of external blood. It can provide a reference for the subsequent genetic improvement of growth traits and early molecularly assisted selection of Dongfeng sika deer.

With the widespread application of molecular biology techniques in livestock breeding, the analysis of genotype combinations in relation to economic traits has improved the accuracy of marker-assisted selection. Zhao [30] reported that individuals carrying the GGAACC genotype combination exhibited superior antler production performance and that the number of favorable genotypes was positively correlated with antler production traits (*p* < 0.01), a finding consistent with results from single-SNP analyses. In the present study, the genotype combination associated with favorable body weight and chest circumference traits was identified as CCCGGC. This finding differs from that of Zhao’s study, which reported that homozygous genotype combinations confer significant advantages for economic traits. The discrepancy between these findings may reflect differences in the analyzed traits, the genetic background of the population, the number and distribution of loci, and management conditions. These observations highlight the complexity of quantitative trait inheritance, where the contributions of individual loci may be influenced by interactions among genes and environmental factors and where the effect of a given genotype may vary depending on the population and trait under consideration.

At present, research on molecular marker-assisted selection in sika deer remains limited, with most studies focusing primarily on antler production performance. Although some functional genes associated with antler development have been identified, relatively few studies have directly addressed growth traits. Recent work by Li [31] and Xue [32] has begun to explore this area, but the genetic basis of growth traits in sika deer remains incompletely understood. Multiple genes with small individual effects typically regulate growth traits, and the contribution of any single gene is often modest. In this study, haplotype block analysis indicated that SNP2 and SNP3 formed a haplotype block (Block 1) with relatively strong linkage disequilibrium, suggesting that these loci are closely linked on chromosome 13 and may be considered jointly in subsequent analyses. Although SNP4 and SNP5 formed a second block (Block 2), the level of association was moderate, which may be influenced by the limited sample size and may not reflect a strong biological relationship. Other loci showed low or negligible linkage, indicating that the selected markers are relatively independent, consistent with the r^2^ analysis results. When considering the combined effects of multiple loci, individuals were classified into three groups based on the number of dominant genotypes. The analysis indicated that individuals with the Type III diplotype (CCCGGC), in which all three loci were dominant, exhibited the highest body weight and chest circumference, whereas individuals with the Type I diplotype, lacking dominant genotypes, exhibited the lowest values. Given that polygenic effects influence growth traits and may exhibit cumulative gene action, this pattern is consistent with expectations for quantitative traits. The identification of the CCCGGC diplotype as a potentially dominant genotype combination suggests it may serve as a useful molecular marker for selecting for growth traits in sika deer, although further validation is required.

Differential fragments need to be verified for expression authenticity using mRNA differential display technology. Internal reference genes such as *GAPDH* are used as controls to compare the expression of target genes in different samples. This technology has the characteristics of high sensitivity, strong specificity, and convenient operation. It has been widely used in functional differential gene expression analysis. Previous studies have utilized this approach to examine gene expression patterns in livestock; for example, Li et al. [33] analyzed the expression of *Plin3* and *Plin5* in pig tissues, while Zhang et al. [34] examined the expression of *IGF-1*, *IGF-2*, and *MSTN* genes in the ear tissue of breeding pigs and their correlation with growth traits. In the present study, significant differences in the relative expression of *GABRA5*, *SOX13*, and *AGL* genes were observed in the blood of six 3-year-old Dongfeng sika deer bucks grouped by body weight and chest circumference. The expression levels of all three genes were lower in the low-growth group compared to the high-growth group, suggesting a positive association between gene expression and growth traits. Within the same group, differences in expression among the three genes were also observed, indicating potential gene-specific regulatory patterns that may be influenced by the phenotypic or physiological state. The relatively lower expression of *AGL* in the low-growth group may suggest a role in metabolic regulation associated with reduced growth performance, whereas the higher expression of *GABRA5* in the high-growth group may indicate a closer association with favorable growth traits and a possible regulatory role in body size development. Further investigation of *GABRA5* and *AGL* may provide additional insight into the molecular mechanisms underlying growth traits and help identify key functional loci for marker-assisted selection in sika deer.

## 5. Conclusions

In this study, an association analysis of *GABRA5*, *SOX13*, and *AGL* gene polymorphism with growth traits of Dongfeng sika deer was carried out. It was found that the coefficient of variation of body weight and chest circumference was high and that body weight was significantly positively correlated with oblique body length and chest circumference. A total of six moderately polymorphic SNP loci conforming to the Hardy–Weinberg equilibrium were identified, and the dominant genotypes were CG (CC), CC (CG), AA, and GG (GC), of which the Chr13-8442730 and Chr13-903380 loci showed strong linkage disequilibrium and CCCGGC was the dominant genotype combination of weight and chest circumference. qRT-PCR verification showed that the expression of three genes was positively correlated with growth traits and that GABRA5 and AGL could be used as candidate genes for molecular breeding. This study can provide scientific reference for efficient molecular breeding, early selection, and genetic improvement of Dongfeng sika deer.

## Figures and Tables

**Table 1:** Primer information list.

Primer Name	Primer Sequence (5′ → 3′)	Primer Sequence (3′ → 5′)	Fragment Size	Annealing Temperature
*GABRA5*	AGGGAGACACTTTAGGCCATTAGAGG	AAGTCCATCCATTGCTACCTGTTCAG	365 bp	61.2 °C
*GABRA5*-2	TTCTCCAAGCCAGGCTTCAG	CTCTTGCTTCCTGACCTGCA	281 bp	57.3 °C
*GABRA5*-3	CACCACCCTTATGGCAGAAAGT	ACCATAGCCTTGACTGGATGGA	335 bp	57.3 °C
*SOX13*	TCAGGGGAGTCTCTCTGCAA	GTGGGATCAGGAGAGACCCT	276 bp	58 °C
*AGL*-1	AATGCACTTTCTGGATGTTCCTGGAG	GCAGAGGACAAGACCCTTCTTAACTC	340 bp	60 °C
*AGL*-2	CAAGCTCTGGGAGTTGGTGA	ACCACCTCTCAAACAGCACT	274 bp	60 °C

**Table 2:** Primer information list for qPCR.

Primer Name	Primer Sequence (5′ → 3′)	Primer Sequence (3′ → 5′)	Fragment Size
*GABRA5*-L	GAAGAAGTCCATCGCCCACA	ATGGTCAAGCGCATGGTGTA	93 bp
*SOX13*-L	ATGAACTGCAGCCTGGAGAC	TGCAAGACAGACTGGACGTC	97 bp
*AGL*-L	TCTTGCTGCAGGCTTACCTC	TGAGGCCATGTCTCAGGGTA	158 bp
*GAPDH*-L	ATCAAGTGGGGTGATGCTGG	GGTTCACGCCCATCACAAAC	157 bp

**Table 3:** Statistics on growth traits of Dongfeng sika deer bucks.

Trait	Body Weight (kg)	Body Height (cm)	Body Length (cm)	Chest Depth (cm)	Chest Circumference (cm)	Head Length (cm)	Frontal Width (cm)	Antler Shank Distance (cm)	Pipe Circumference (cm)	Tail Length (cm)
Mean ± SD	117.13 ± 26.08	105.67 ± 4.98	108.50 ± 5.87	47.13 ± 3.08	120.86 ± 9.03	30.76 ± 3.13	13.02 ± 0.91	10.08 ± 0.97	10.07 ± 0.81	15.42 ± 2.20
Coefficient of variation	22.26	4.71	5.41	6.54	7.47	10.17	6.96	9.60	7.57	13.10

**Table 4:** Correlation coefficient matrix of growth traits of Dongfeng sika deer bucks.

Trait	Y	X_1_	X_2_	X_3_	X_4_	X_5_	X_6_	X_7_	X_8_	X_9_
Y	1									
X_1_	0.744 **	1								
X_2_	0.326 **	0.513 **	1							
X_3_	0.855 **	0.689 **	0.511 **	1						
X_4_	0.282 **	0.158	0.184	0.362 **	1					
X_5_	0.208 *	0.099	0.235 **	0.287 **	0.033	1				
X_6_	0.529 **	0.468 **	0.424 **	0.550 **	0.107	0.266 **	1			
X_7_	0.443 **	0.279 **	0.320 **	0.380	0.264 *	0.173 **	0.265 **	1		
X_8_	0.080	0.055	0.130	0.109	0.065	0.018	−0.065	0.040	1	
X_9_	0.176	0.355 **	0.215 *	0.209 *	−0.044	0.068	0.249 *	0.034	0.061	1

Note: Y–X_9_ represent body weight (WT), body length (BL), body height (HT), chest circumference (WS), chest depth (CC), head length (HL), frontal width (FW), pipe circumference (PC), tail length (WC), and antler shank distance (JB), respectively; * indicates a significant correlation (*p* < 0.05); ** indicates an extremely significant correlation (*p* < 0.01).

**Table 5:** Loci of SNPs associated with some growth traits.

Trait	Chromosome	Location, bp	Genotype	*p*-Value	Proximity Gene
Body Weight	Chr12	12441747	A (T)	1.07 × 10^−13^	*NRXN3*
	Chr13	8442730	C (G)	11.69 × 10^−23^	*GABRA5*
	Chr14	5681678	C (G)	7.95 × 10^−23^	*SOX13*
	Chr20	66618510	G (C)	8.77 × 10^−23^	*AGL*
	Chr14	2063506	A (G)	2.94 × 10^−19^	*LOC122708028*
	Chr24	52547833	A (T)	9.93 × 10^−17^	*FHIT*
	Chr24	55874319	G (C)	1.81 × 10^−19^	*ERC2*
Chest Circumference	Chr12	10590680	C (G)	11.29 × 10^−23^	*TSHR*
	Chr12	10599421	A (T)	7.67 × 10^−23^	
	Chr13	8442730	C (G)	8.95 × 10^−23^	*GABRA5*
	Chr13	9033380	A (T)	7.25 × 10^−23^	
	Chr13	9045819	C (G)	7.49 × 10^−23^	
	Chr14	5681678	C (G)	8.62 × 10^−23^	*SOX13*
	Chr20	66603370	A (T)	7.39 × 10^−23^	*AGL*
	Chr20	66618510	G (C)	12.91 × 10^−23^	
	Chr20	65299752	A (C)	22.78 × 10^−23^	*S1PR1*, *OLFM3*
	Chr23	55367590	T (A)	8.98 × 10^−23^	*CDH4*
	Chr28	13896680	A (T)	13.87 × 10^−23^	*RISM1*
	Chr28	13896685	A (T)	17.07 × 10^−23^	
	Chr28	13896686	C (G)	10.55 × 10^−23^	
	Chr28	13896700	G (C)	12.86 × 10^−23^	
	Chr28	13896701	G (C)	12.86 × 10^−23^	
	Chr28	13896704	C (G)	14.44 × 10^−23^	
	Chr28	852575	T (C)	20.38 × 10^−23^	*LOC122685417*
	Chr29	716609	T (C)	24.64 × 10^−23^	*LOC122686330*

**Table 6:** SNP site information.

Locus	Genotype	Genotype Frequency	Allele	Allele Frequency
Chr13 8442730	CC	0.833	C	0.917
	CG	0.167	G	0.083
Chr13 9033380	AA	0.667	A	0.833
	AT	0.333	T	0.167
Chr13 9045819	CC	0.917	C	0.958
	CG	0.083	G	0.042
Chr14 5681678	CC	0.444	C	0.722
	CG	0.556	G	0.278
Chr20 66603370	AA	0.750	A	0.875
	AT	0.250	T	0.125

**Table 7:** Genetic parameters of 6 SNP loci in growth traits of the Sika deer population.

Locus	PIC	Ho	He	Ne	MAF	HWE *p*-Value	Gene
Chr13 8442730	0.233	0.167	0.278	1.180	0.167	*p* > 0.05	*GABRA5*
Chr13 9033380	0.345	0.333	0.444	1.385	0.333		
Chr13 9045819	0.142	0.083	0.153	1.087	0.083		
Chr14 5681678	0.375	0.500	0.500	1.670	0.500	*p* > 0.05	*SOX13*
Chr20 66603370	0.302	0.250	0.375	1.280	0.250	*p* > 0.05	*AGL*
Chr20 66618510	0.280	0.222	0.346	1.246	0.222		

Note: PIC, polymorphic information content; Ho, observed heterozygosity; He, desired heterozygosity; Ne, number of effective alleles; MAF, minimal allele; HWE *p*-value, *p*-value of the Hardy–Weinberg Equilibrium.

**Table 8:** Multiple comparisons of 6 SNP genotypes significantly associated with bodyweight and chest circumference traits.

Locus	Genotype	Count	Genotype Frequency	Mean Body Weight (kg)	Mean Chest Circumference (cm)
Chr13 8442730	CC	30	0.833	99.94 ± 14.14 ^a^	110.67 ± 7.95 ^a^
	CG	6	0.167	86.05 ± 12.85 ^b^	122.33 ± 10.23 ^b^
Chr13 9033380	AA	24	0.667	-	120.33 ± 8.68 ^a^
	AT	12	0.333	-	112.67 ± 7.83 ^b^
Chr13 9045819	CC	33	0.917	-	113.33 ± 8.34 ^a^
	CG	3	0.833	-	124.97 ± 4.16 ^b^
Chr14 5681678	CC	16	0.444	85.93 ± 13.52 ^a^	119.25 ± 8.06 ^a^
	CG	20	0.556	99.48 ± 14.61 ^b^	107.80 ± 8.80 ^b^
Chr20 66603370	AA	27	0.750	-	120.81 ± 8.74 ^a^
	AT	9	0.250	-	109.33 ± 7.62 ^b^
Chr20 66618510	GG	28	0.778	87.12 ± 14.13 ^a^	119.29 ± 8.24 ^a^
	GC	8	0.222	100.73 ± 15.05 ^b^	107.50 ± 8.80 ^b^

Note: The same superscript letter in the same column indicates that the difference is not significant, and the difference between the values of different superscript letters is significant (*p* < 0.05).

**Table 9:** Linkage disequilibrium analysis (D’ and r^2^ values) of 6 SNPs in *GABRA5*, *SOX13*, and *AGL* genes of sika deer.

SNPs	SNP1	SNP2	SNP3	SNP4	SNP5	SNP6
SNP1	-	0.98 *	0.85 *	0.00	0.00	0.00
SNP2	0.41 **	-	0.92 *	0.00	0.00	0.00
SNP3	0.23 **	0.32	-	0.00	0.00	0.00
SNP4	0.24 **	0.32	0.09	-	0.00	0.00
SNP5	0.16 **	0.20	0.06	0.32	-	0.07 **
SNP6	0.07 **	0.16	0.06	0.20	0.32	-

Note: The upper triangle is the D’ value, and the lower triangle is the r^2^ value. * D’ > 0.8 is a strong chain, and ** r^2^ > 0.33 is a strong chain. SNP1-SNP3 is the *GABRA5* gene locus, SNP4 is the *SOX13* gene locus, and SNP5-SNP6 is the *AGL* gene locus.

**Table 10:** 3 SNP loci in different genotypic combinations of bodyweight traits.

Type	Number of Dominant Genotypes	Number of Individuals	Genotypic Combination	Mean Body Weight (kg)	Mean Chest Circumference (cm)
Type I	0	4	CGCCGC	81.51 ± 15.67 ^a^	102.62 ± 7.67 ^a^
			CGCCGG		
Type II	1 or 2	27	CCCCGC	94.97 ± 13.14 ^b^	115.27 ± 4.82 ^b^
			CCCCGG		
			CCCGGC		
			CGCGGG		
Type III	3	5	CCCGGC	110.44 ± 16.98 ^c^	128.95 ± 13.20 ^c^

Note: The genotype combination is carried out according to the positions of SNP 13-8442730, SNP 14-568167, and SNP 20-66618510 in sequence. The same superscript letter in the same column indicates that the difference is not significant, and the difference between the values of different superscript letters is significant (*p* < 0.05).

**Table 11:** Statistics of body weight and chest circumference of Dongfeng sika deer bucks in different groups.

Trait	Group	Maximum	Minimum	Mean	Standard Deviation	Coefficient of Variation
Body weight (kg)	Low	82.4	72.6	76.2	5.39	0.07
	High	97	94.6	95.73	1.21	0.01
Chest circumference (cm)	Low	120	102	114.93	4.83	0.04
	High	140	122	126.13	5.15	0.04

## Data Availability

All data generated or analyzed during this study are included in this published article. Due to animal ethical privacy restrictions, the original raw experimental data cannot be publicly shared. All data supporting the conclusions of this study can be obtained from the corresponding author upon reasonable request.

## Supplementary Materials

The following supporting information can be downloaded at: https://www.mdpi.com/article/10.3390/biology15110881/s1.
