# Supplementary figures and images for: Association Analysis of GABRA5, SOX13, and AGL Gene Polymorphisms with Growth Traits in Dongfeng Sika Deer
Source: Biology (Basel). 2026 Jun 3;15(11):881. doi: 10.3390/biology15110881 (PMC13255946; doi:10.3390/biology15110881)

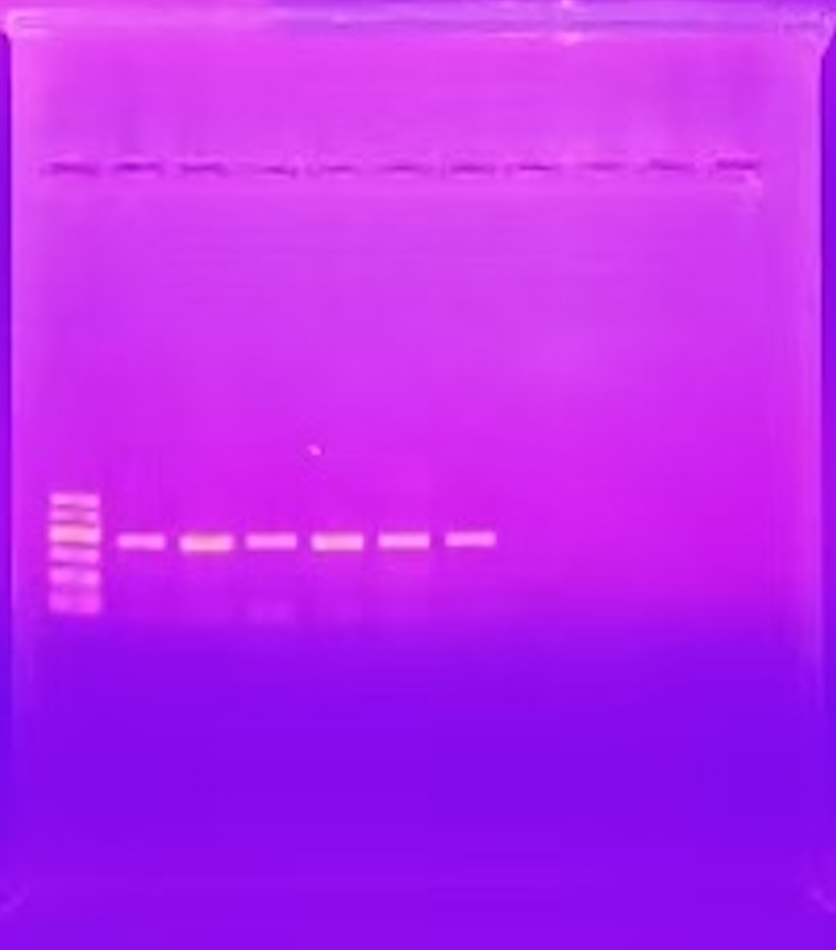

Supplement: Supplementary file 1 [file biology-15-00881-s001.zip › S1-GABRA5 Agarose gel electrophoresis diagram.jpg]

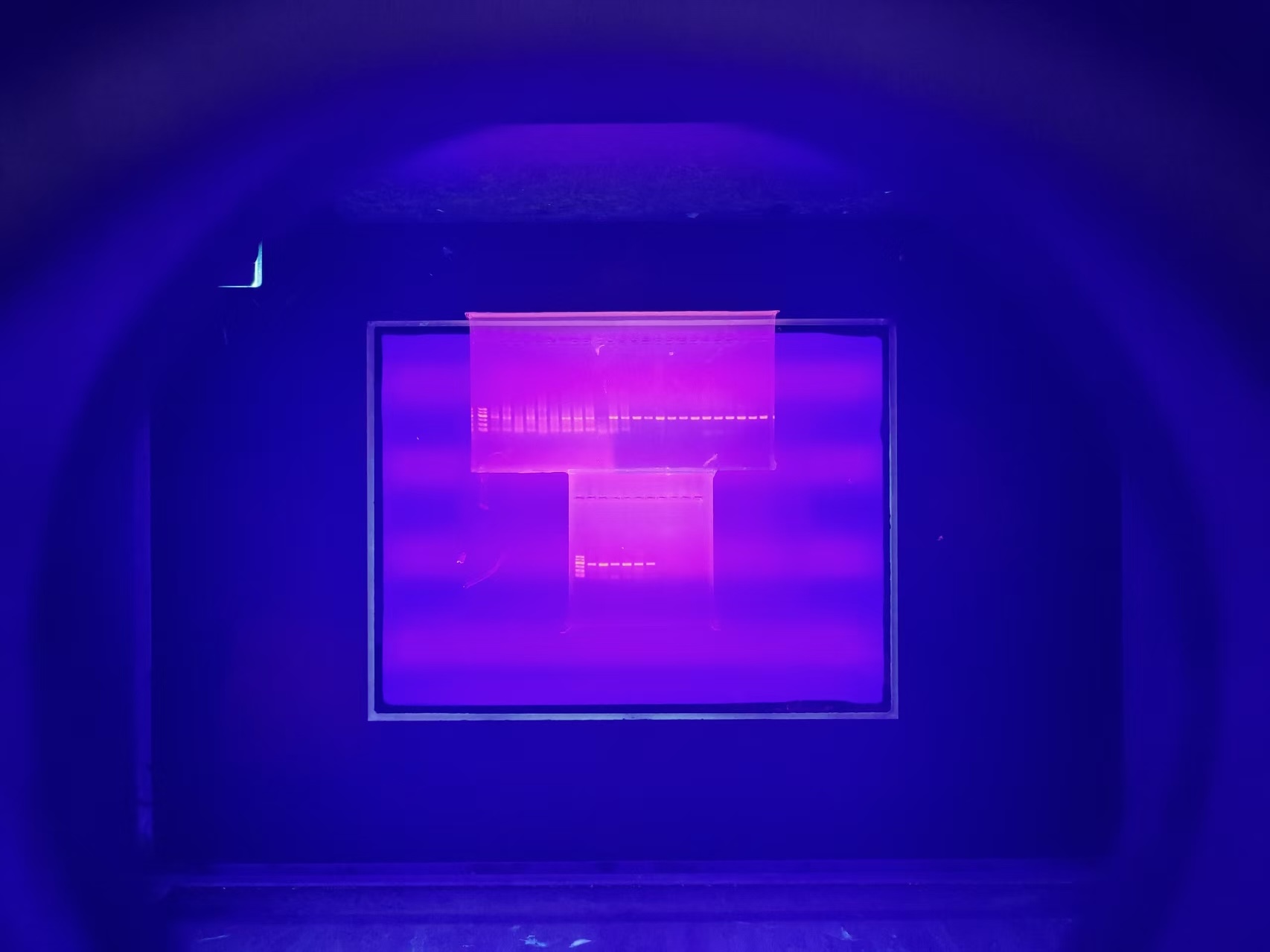

Supplement: Supplementary file 1 [file biology-15-00881-s001.zip › S1-GABRA5.jpg]

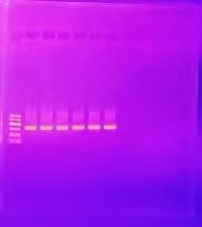

Supplement: Supplementary file 1 [file biology-15-00881-s001.zip › S2-GABRA5-2 Agarose gel electrophoresis diagram.jpg]

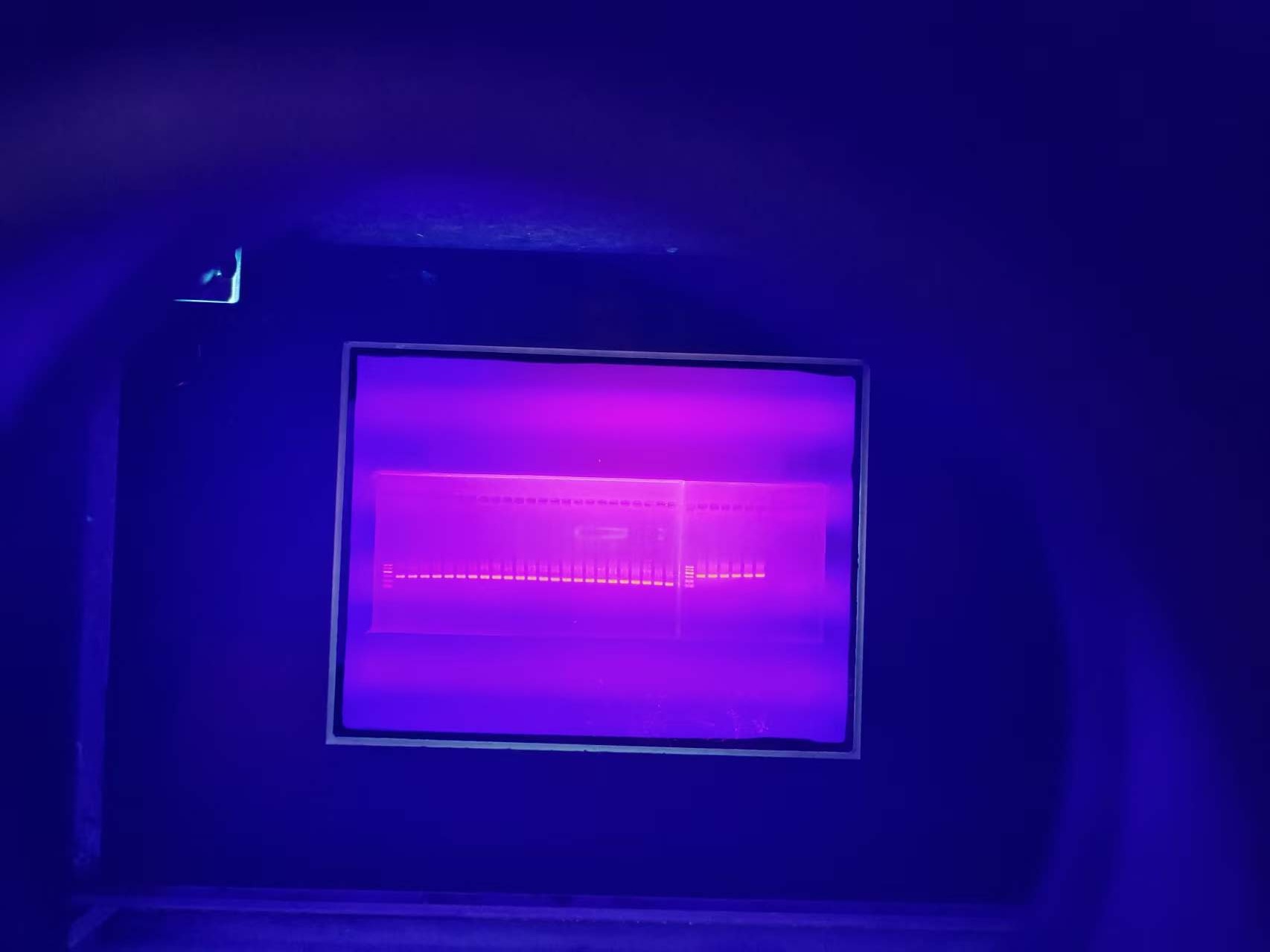

Supplement: Supplementary file 1 [file biology-15-00881-s001.zip › S2-GABRA5-2.jpg]

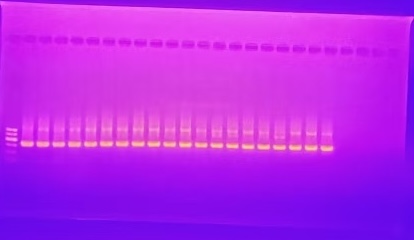

Supplement: Supplementary file 1 [file biology-15-00881-s001.zip › S3-GABRA5-3 Agarose gel electrophoresis diagram.jpg]

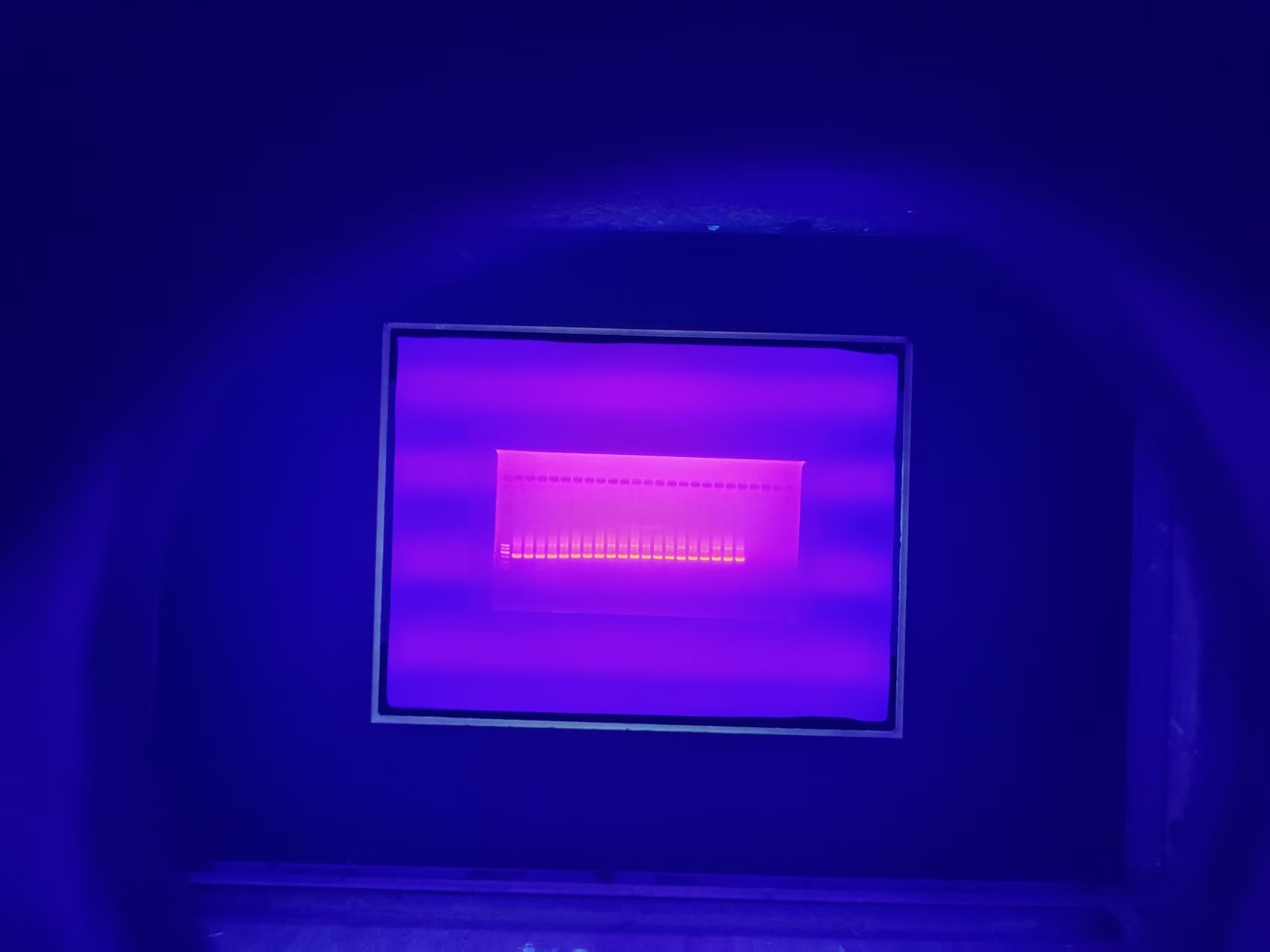

Supplement: Supplementary file 1 [file biology-15-00881-s001.zip › S3-GABRA5-3.jpg]

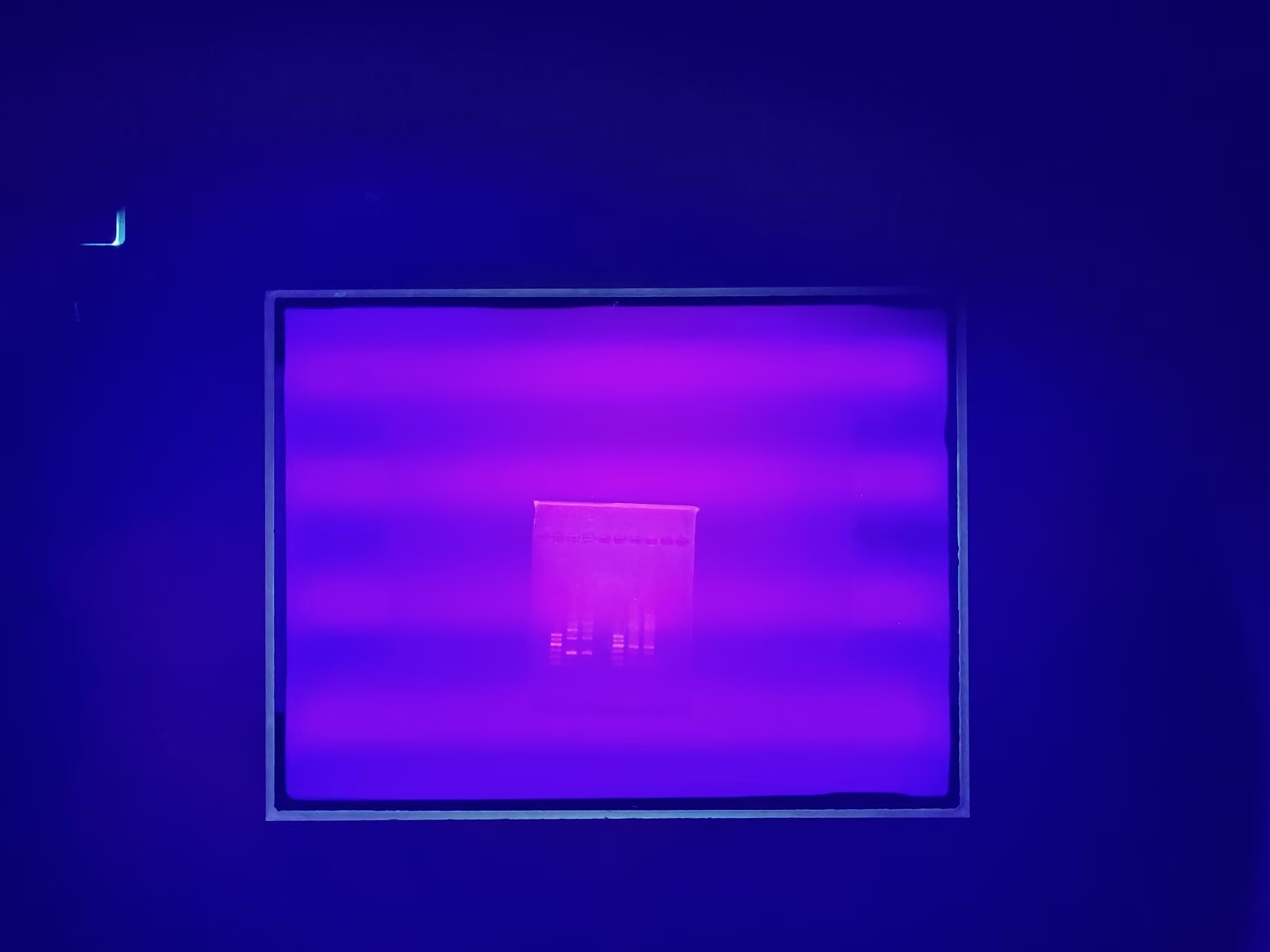

Supplement: Supplementary file 1 [file biology-15-00881-s001.zip › S4 and S6-SOX13、AGL-2.jpg]

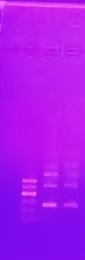

Supplement: Supplementary file 1 [file biology-15-00881-s001.zip › S4-SOX13 Agarose gel electrophoresis diagram.jpg]

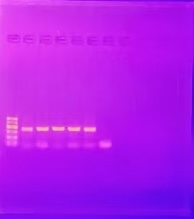

Supplement: Supplementary file 1 [file biology-15-00881-s001.zip › S5-AGL-1 Agarose gel electrophoresis diagram.jpg]

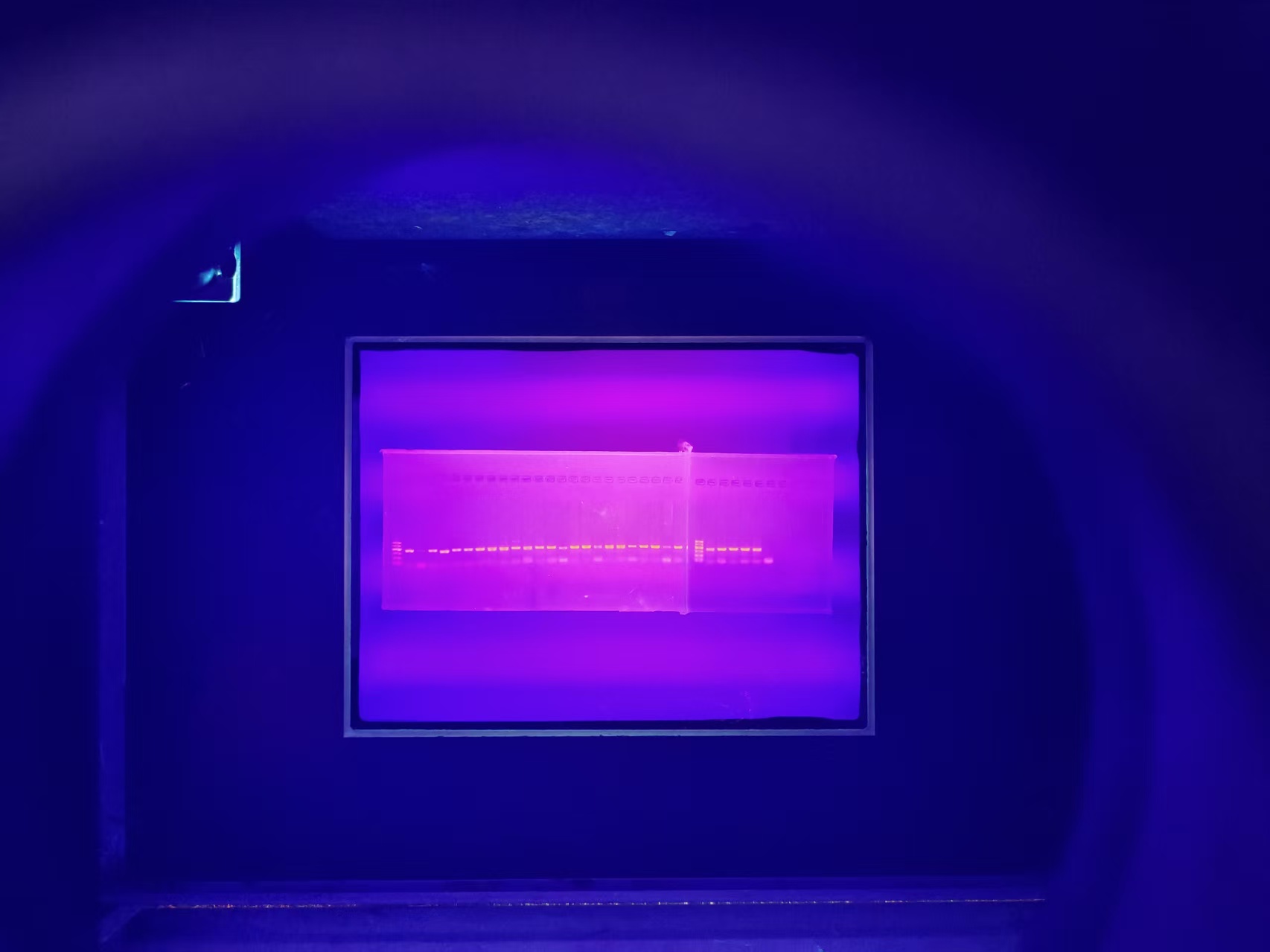

Supplement: Supplementary file 1 [file biology-15-00881-s001.zip › S5-AGL-1.jpg]

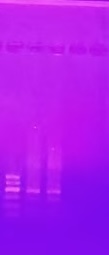

Supplement: Supplementary file 1 [file biology-15-00881-s001.zip › S6-AGL-2 Agarose gel electrophoresis diagram.jpg]
